# The Discovery of the Monoaminergic Innervation of the Cerebellum: Convergence of Divergent and Point-to-Point Systems

**DOI:** 10.1007/s12311-022-01480-7

**Published:** 2022-09-23

**Authors:** Lazaros C. Triarhou, Mario Manto

**Affiliations:** 1 Department of Psychology, Sector of Experimental Cognitive Psychology, Aristotelian University Faculty of Philosophy, University Campus, 54124 Thessaloniki, Greece; 2 Unité Des Ataxies Cérébelleuses, CHU-Charleroi, Charleroi, Belgium; 3 Service Des Neurosciences, University of Mons, Mons, Belgium

**Keywords:** Cerebellar afferents, Monoamines, Norepinephrine, Serotonin, Developmental plasticity

## Abstract

This *Cerebellar Classic* highlights the landmark discovery of the innervation of the cerebellar cortex and cerebellar nuclei by noradrenergic and serotoninergic axons emanating, respectively, from the locus coeruleus and the raphé nuclei. Since then, modulation of the activity of cerebellar neurons by the monoamine systems has been studied extensively, as well as their reorganization and modifications during development, plasticity, and disease. The discovery of noradrenergic and serotoninergic innervation of the cerebellum has been a crucial step in understanding the neurochemical relationships between brainstem nuclei and the cerebellum, and the attempts to treat cerebellar ataxias pharmacologically. The large neurochemical repertoire of the cerebellum represents one of the complexities and challenges in the modern appraisal of cerebellar disorders.

Divergent or “global” neural systems imply situations, whereby a relatively small number of neurons innervate a much larger number of terminal domains, in contrast to so-called “point-to-point” systems, where each neuron only contacts a few target nerve cells [1]. Since the time of Ramón y Cajal, the cerebellum had classically been considered a point-to-point system. With the discovery in the 1960s of the cerebellar monoaminergic innervation by neurons of the locus coeruleus and the raphé nuclei, the cerebellum has become a structure where “point-to-point” and “global” neural circuits converge. Moreover, monoamines may exert a widespread effect on neurons besides those receiving physical synaptic appositions—that is, they may subserve a paracrine function [2].

In the human brain, the locus coeruleus contains an average of 50,000 noradrenergic neurons [3, 4], while in rodents it contains about 3,000 cells [5]. The dorsal raphé nuclei of the human brain contain around 130,000–200,000 serotoninergic neurons [6], while that number in rodents is about 8,000–9,000 cells [7, 8]. Thus, the thousands of neurons in both these anatomical systems influence the physiological activity of extensively divergent domains that comprise several billion neurons, from the telencephalon to the spinal cord.

The *Cerebellar Classic* [9] by the pioneer Swedish neuroscientists Nils-Erik Andén, Kjell Fuxe, and Urban Ungerstedt revisited here has broadened the sources of afferent input to the cerebellum beyond the “traditional” climbing and mossy fibers. It has also paved the way for studies on the fate and reorganization of cerebellar monoamine systems in human diseases [10] and in experimental models of cerebellar degeneration [11–21], as well the elucidation of phylogenetic [22, 23], ontogenetic [24–26], developmental plasticity [27–29], and reinnervation issues [30].

Andén and colleagues [9] studied central monoamine neurons and their unmyelinated axons by means of fluorescence histochemistry after removing the cerebral cortex and cerebellum by suction with a fine glass cannula. In biochemical measurements, they found the mean concentration of norepinephrine in the normal rat cerebellum to be 0.18 μg/g, representing approximately 8% of the total brain amount; the mean concentration of serotonin was 0.07 μg/g or about 2.5% of the total brain amount. The authors concluded that most, if not all, norepinephrine nerve terminals in the cerebral cortex and the cerebellum belonged to axons originating from noradrenergic cell bodies primarily located in the reticular formation of the medulla oblongata and the pons. They further articulated the idea that the same noradrenergic neuron may innervate both the cerebral cortex and the cerebellum. That last organizing principle of the anatomical projections of coerulear noradrenergic neurons was subsequently confirmed with the identification of collateral axons in the cerebellar cortex, the cerebellar nuclei, and other areas of the central nervous system, including the cerebral cortex, the diencephalon, and the spinal cord [31, 32].

The monoaminergic innervation of the cerebellar cortex comprises norepinephrine- and serotonin-containing axons (Fig. 1) [9, 31]. The origin of the noradrenergic projection lies in neurons of the dorsal part of the locus coeruleus [33–35], the nucleus subcoeruleus, and fields A5/A7 [22, 36, 37]. Furthermore, horseradish peroxidase (HRP) tracing experiments in rats showed heavy innervation of the locus coeruleus by all raphé nuclei, in addition to many extra-raphé brainstem sources [38], suggesting close interactions between the two main brainstem monoaminergic nodes that target the cerebellar circuitry. Using fluorescence histochemistry, researchers have found that the noradrenergic innervation of the cerebellar cortex is more pronounced than its serotoninergic innervation [31]. Electron microscopic studies have shown that, in the rodent cerebellum, norepinephrine-containing axons are apposed to Purkinje cell dendrites [39, 40].

**Fig. 1:**
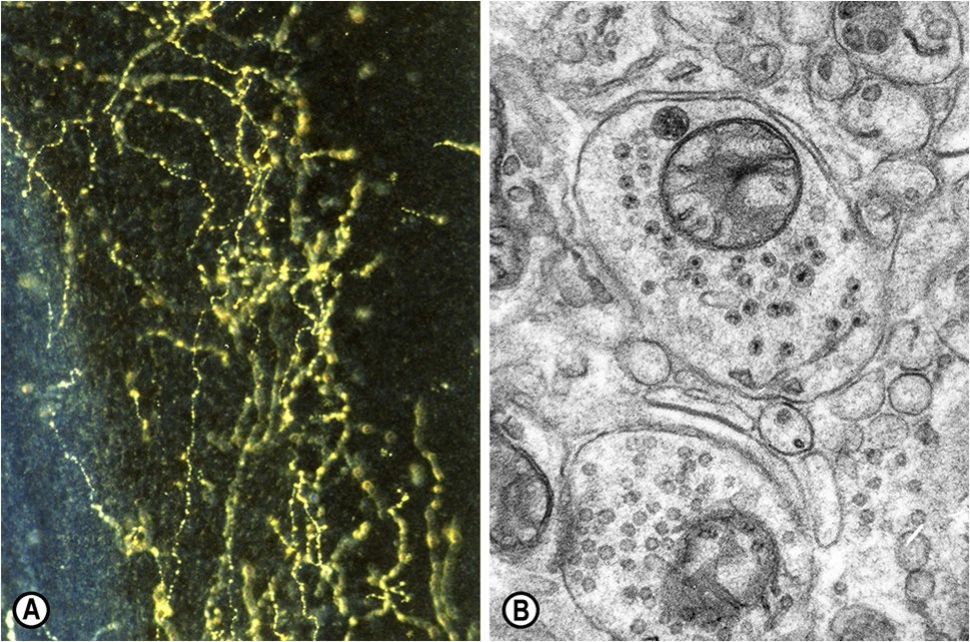
(**A**) Serotonin-immunoreactive fibers in the mouse cerebellum, displaying their typical axonal varicosities. Sternberger peroxidase-antiperoxidase (PAP) method, dark-field illumination, × 40 [18]. (**B**) Electron micrograph of a monoaminergic varicosity or bouton *en passant* in the molecular layer of the cerebellum of a “Purkinje cell degeneration” (*Agtpbp1*^*pcd*^/*Agtpbp1*.^*pcd*^) mutant mouse, containing small granular vesicles (40–60 nm in diameter) and a large granular vesicle (90–110 nm in diameter). Potassium permanganate (KMnO_4_) fixation method, ultrathin section stained with uranyl acetate and lead citrate, × 24,000 [16]

Physiological experiments have indicated a neuromodulatory role for norepinephrine [41, 42] and serotonin [43], both adjusting the activity of other synaptic inputs to the Purkinje cells rather than exerting a strict excitatory or inhibitory effect. The presence of *α* and *β* adrenergic receptors on Purkinje cells suggests the existence of bidirectional mechanisms of regulation that allow noradrenergic afferents to refine the signals arriving at Purkinje cells, including the parallel fiber input, under specific arousal states or during motor skill learning [44]. Cerebellar catecholamines, especially in the lateral cerebellar nucleus, might modulate certain aspects of cognitive and affective behavior, such as sensorimotor integration, associative fear learning, response inhibition, and working memory [45].

Serotonin-containing axons originate in neurons of the dorsal raphé nuclei of the pons and of the medullary and pontine reticular formation [46–48], and are distributed throughout the cerebellar cortex of the rat [9, 31, 48, 49] and the mouse [18]. A small contingent of serotonin terminals belong to typical mossy fibers; these are confined to the granule cell layer and establish synapses on dendrites of granule cells [24, 50]. The vast majority of serotonin nerve terminals belong to finer beaded axons of the so-called “diffuse system” and are distributed to all cerebellar cortical layers [50]. Serotonin axon terminals innervate the dendrites of Purkinje and granule cells; the parallel fibers; as well as basket, stellate, and Golgi cells and neurons of the cerebellar nuclei [24, 46, 48, 50]. Iontophoretic application of serotonin and electrophysiological stimulation of the raphé nuclei modulate the firing of Purkinje cells [43, 51–53]. Moreover, serotonin modulates the glutamate-induced excitation and the *γ*-aminobutyric acid (GABA)-elicited inhibition of Purkinje cells [54, 55].

With regard to the “third monoamine,” dopamine (3,4-dihydroxyphenethylamine), the cerebellum had not been considered an elective dopaminergic region, and the very small amounts of dopamine detected in it were thought to represent an intermediary product in the metabolism of norepinephrine [56]. Later studies have suggested the presence of a small dopaminergic contingent in the cerebella of rodents and primates [57–59], as well as the expression of dopamine D_1_–D_5_ receptors and dopamine transporters [21]. Still, the density of dopamine D_2_ receptors in the cerebellum represents about 1% of their density in the striatum [60]. Although unequivocal evidence on the functional role of a cerebellar dopaminergic system is still lacking, its involvement in associative and projective circuits has been discussed [61].

This *Cerebellar Classic* highlights a milestone in the elucidation of the neurochemistry of the cerebellum, whose main transmitters and neuromodulators also include glutamate, GABA, acetylcholine, nitric oxide, endocannabinoids, and neuropeptides. This large neurochemical arsenal is one the features of the cerebellum; they are involved in the numerous motor/non-motor functions of the cerebellum and have variable impacts on cerebellar ataxias.
